# Patterns and severity of vascular amyloid in Alzheimer’s disease associated with duplications and missense mutations in APP gene, Down syndrome and sporadic Alzheimer’s disease

**DOI:** 10.1007/s00401-018-1866-3

**Published:** 2018-05-16

**Authors:** David M. A. Mann, Yvonne S. Davidson, Andrew C. Robinson, Nancy Allen, Tadafumi Hashimoto, Anna Richardson, Matthew Jones, Julie S. Snowden, Neil Pendleton, Marie-Claude Potier, Annie Laquerrière, Vee Prasher, Takeshi Iwatsubo, Andre Strydom

**Affiliations:** 1 Division of Neuroscience and Experimental Psychology, Faculty of Biology, Medicine and Health, School of Biological Sciences, Salford Royal Hospital, University of Manchester, Salford, UK; 2 Department of Neuropathology, Graduate School of Medicine, University of Tokyo, Tokyo, Japan; 3 Cerebral Function Unit, Greater Manchester Neurosciences Centre, Salford Royal Hospital, Stott Lane, Salford, UK; 4 ICM Institut du Cerveau et de la Moelle épinière, CNRS UMR7225, INSERM U1127, UPMC, Hôpital de la Pitié-Salpêtrière, 47 Bd de l’Hôpital, Paris, France; 5 Department of Pathology, Rouen University Hospital, Rouen, France; 6 Normandie Univ, UNIROUEN, CHU Rouen, INSERM U1245, Team 4, Neovasc, 76000 Rouen, France; 7 Birmingham Community NHS Trust, The Greenfields, 30 Brookfield Road, Birmingham, B30 3QY UK; 8 Institute of Psychiatry, Psychology and Neuroscience, King’s College London, 16 De Crespigny Park, London, UK; 9 Division of Psychiatry, University College London, 147 Tottenham Court Road, London, UK

**Keywords:** Alzheimer’s disease, Down syndrome, *APP* mutations, Cerebral amyloid angiopathy, Amyloid plaques

## Abstract

In this study, we have compared the severity of amyloid plaque formation and cerebral amyloid angiopathy (CAA), and the subtype pattern of CAA pathology itself, between *APP* genetic causes of AD (*APP*dup, *APP* mutations), older individuals with Down syndrome (DS) showing the pathology of Alzheimer’s disease (AD) and individuals with sporadic (early and late onset) AD (sEOAD and sLOAD, respectively). The aim of this was to elucidate important group differences and to provide mechanistic insights related to clinical and neuropathological phenotypes. Since lipid and cholesterol metabolism is implicated in AD as well as vascular disease, we additionally aimed to explore the role of *APOE* genotype in CAA severity and subtypes. Plaque formation was greater in DS and missense *APP* mutations than in *APP*dup, sEOAD and sLOAD cases. Conversely, CAA was more severe in *APP*dup and missense *APP* mutations, and in DS, compared to sEOAD and sLOAD. When stratified by CAA subtype from 1 to 4, there were no differences in plaque scores between the groups, though in patients with *APP*dup, *APP* mutations and sEOAD, types 2 and 3 CAA were more common than type 1. Conversely, in DS, sLOAD and controls, type 1 CAA was more common than types 2 and 3. *APOE* ε4 allele frequency was greater in sEOAD and sLOAD compared to *APP*dup, missense *APP* mutations, DS and controls, and varied between each of the CAA phenotypes with *APOE* ε4 homozygosity being more commonly associated with type 3 CAA than types 1 and 2 CAA in sLOAD and sEOAD. The differing patterns in CAA within individuals of each group could be a reflection of variations in the efficiency of perivascular drainage, this being less effective in types 2 and 3 CAA leading to a greater burden of CAA in parenchymal arteries and capillaries. Alternatively, as suggested by immunostaining using carboxy-terminal specific antibodies, it may relate to the relative tissue burdens of the two major forms of Aβ, with higher levels of Aβ_40_ promoting a more ‘aggressive’ form of CAA, and higher levels of Aβ_42(3)_ favouring a greater plaque burden. Possession of *APOE* ε4 allele, especially ε4 homozygosity, favours development of CAA generally, and as type 3 particularly, in sEOAD and sLOAD.

**Electronic supplementary material:**

The online version of this article (10.1007/s00401-018-1866-3) contains supplementary material, which is available to authorized users.

## Introduction

Alzheimer’s disease (AD) is a neurodegenerative disorder characterised clinically by a progressive loss of memory and cognition, accompanied by functional impairments of orientation and praxis. Pathologically, the major changes involve a deposition of amyloid β protein (Aβ) in brain parenchyma (as amyloid plaques) and hyperphosphorylated tau within neurones (as neurofibrillary tangles). Additionally, most cases display deposits of Aβ within blood vessel walls—a change known as cerebral amyloid angiopathy (CAA). While more than 90% cases of AD are without obvious genetic cause, and termed ‘sporadic’, the remainder is associated with mutational events involving either the Amyloid Precursor Protein (*APP*) or presenilin (*PSEN*) genes.

With respect to the transmembrane protein *APP*, missense mutations changing the amino acid sequence at either the amino- or carboxy-terminal points of the Aβ sequence (e.g. *APP*670/671, *APP*717) result in increased catabolic breakdown of APP by β- and/or γ-secretase into Aβ, and confer a pathological picture similar to that seen in sporadic AD. On the other hand, mutations lying in the juxtamembrane region, such as *APP*692 or *APP*693, are more associated with CAA than plaques, and often manifest clinically as acute stroke. There are also rare French [6, 14, 37], Dutch [41], Finnish [38], Japanese [19], Swedish [48] and British [30] families where AD is linked to duplications at the *APP* locus, resulting in APP overproduction. In most of these families, the duplication has been validated only in living patients and confirmed cases with brain donation are scarce. An *APP* duplication has also been reported in a Spanish patient with apparently sporadic AD and severe CAA [21], but other studies of sporadic AD with CAA have not identified such duplications [3, 11]. It has long been known that most individuals with Down syndrome (DS), who live into middle age and beyond, show a pathological picture indistinguishable from that of AD [24, 25]. In most DS individuals, there is a complete triplication of chromosome 21, including the *APP* locus. In both *APP*dup and DS individuals, it is presumed that the early deposition of Aβ plaques and CAA stems from an overexpression of *APP* and the consequent degradation of an excessive production of APP. In addition, recent work suggests that a mutation in the 3′untranslated region of *APP* also result in APP overexpression and might act as a genetic determinant in some cases of CAA [33].

Although all cases of AD are defined pathologically by the presence of numerous plaques and tangles, and usually CAA, throughout the cerebral cortex and hippocampus, the morphological appearance of these changes, especially plaques and CAA, can vary according to the underlying genetic background. For example, a much more severe CAA is seen in patients with presenilin-1 (*PSEN*-*1*) mutations located beyond codon 200 compared to those where the mutation lies before codon 200 [28]. Also, certain *PSEN*-*1* mutations are associated with an unusual morphological form of amyloid plaques, known as ‘cotton wool’ plaques, and often present clinically with a spastic paresis [29].

The cardinal clinical presentation in *APP* duplications (*APP*dup) is that of progressive dementia frequently accompanied by seizures and intracerebral haemorrhage (ICH) [6, 14, 19, 37, 38, 41], and neuropathological studies have revealed severe CAA in association with abundant Aβ plaques and neurofibrillary tangles, and occasionally Lewy bodies [6, 14]. CAA is also often prominent in individuals with DS [25] where there is also an additional *APP* copy number. Nevertheless, CAA can present in sporadic and familial AD in various phenotypic histological forms, involving differing combinations of leptomeningeal and parenchymal arterial pathology, sometimes extending into the capillary bed [2, 46].

We have recently reviewed epidemiological data on stroke (including haemorrhagic stroke) related to CAA to make comparisons between DS and *APP*dup [5]. Although ICH, the main clinical consequence of vascular amyloidosis, is a common clinical occurrence in *APP*dup, this is a more poorly defined feature of individuals with DS, suggesting the presence of a mechanism(s) that acts protectively [5]. This might seem somewhat paradoxical given that DS only differs from *APP*dup in the ~ 270 other genes located on chromosome 21 that are also triplicated.

However, a direct comparison of vascular amyloidosis at pathological level between DS, *APP*dup, and other *APP* mutations has never been undertaken as far as we are aware, and consequently the degree and nature of tissue differences in CAA, ICH, and Aβ deposition between these disorders remain unclear. Therefore, in this analysis, we aim to compare the severity of amyloid plaque formation and CAA, and the subtype pattern of CAA pathology itself, and the relationship between CAA severity and CAA phenotype, in *APP* genetic causes of AD (*APP*dup, *APP* mutations), DS and sporadic (early and late onset) AD (sEOAD and sLOAD, respectively). The observations might help to elucidate important differences between the patient groups, and thus provide mechanistic insights related to clinical and neuropathological phenotypes. Since lipid and cholesterol metabolism is implicated in AD as well as vascular disease, we additionally aimed to explore the role of *APOE* genotype in CAA severity and subtypes.

## Materials and methods

### Patients

The study included 152 subjects in total, comprising six groups categorised according to the pathological and genetic basis of their condition. First, brains were obtained from four patients (three males, one female, patients #1–4) with genetically proven duplications in *APP* gene through Prof Annie Laquerrière at University of Rouen. These were from two separate families. Three patients (patients #1–3) were members of the same family (known as F037, and identified as II-4, II-5 and II-6, respectively, in [6]). The other patient (patient #4) was a member of a second family (identified as II.1 in [14]). Second, brains were obtained from 34 individuals with DS (21 males, 13 females, patients #5–38) ranging (at death) from 36 to 69 years. Where karyotyping had been performed, all were full trisomy 21. Eight of these were drawn from the Manchester Brain Bank (MBB), 4 were obtained from the Neurodegenerative Diseases Brain Bank at Institute of Psychiatry, Psychology and Neuroscience (IOPPN) Brain Bank, London (courtesy of C Troakes), 8 from the Thomas Willis Brain Bank (TWBB), Oxford (courtesy of M Esiri), with the remaining 14 being obtained through Professor V P Prasher at University of Birmingham. Third, brains were obtained from 16 patients (seven males, nine females, patients #39–54) with missense mutations in *APP* gene, 14 with point mutations at codon 717 (10 Val717Ile, 3 Val717Gly and 1 Val717Ala) and 2 with a point mutation at codon 692 (Flemish mutation). Four of these were obtained through MBB, eight from IOPPN Brain Bank (courtesy of C Troakes), and four from Queen Square Brain Bank (QSBB) (courtesy of L Parsons). Additionally, brains were obtained from 34 patients with clinical diagnosis of sEOAD (21 males, 13 females, patients #55–88), 34 with sLOAD (18 males, 16 females, patients #89–122) and 30 elderly controls (11 males, 19 females, subjects #123–152) through MBB (see Table 1 for group, and Supplementary Table 1 for individual, patient details). All brains had been obtained at autopsy through appropriate consenting procedures with Local Ethical Committee approval. Patients #1–4 and #39–122 all fulfilled relevant pathological diagnostic criteria for AD [16, 31, 32]. The sEOAD patients acquired through MBB had been investigated longitudinally within specialist dementia clinics at Salford Royal Hospital using the Manchester Neuropsychological Profile (Man-NP) [42, 47] to determine and characterise the nature of their dementia. The sLOAD patients and elderly controls were drawn partly from the Manchester recruits for the Brains for Dementia Research (BDR) cohort, and partly from The University of Manchester Longitudinal Study of Cognition in Normal Healthy Old Age [36]. The 14 individuals with DS who had been assessed clinically by Professor V P Prasher at University of Birmingham had all been diagnosed as suffering from non-familial ‘Dementia in Alzheimer’s disease’ according to DCR-10 criteria [51]. This kind of clinical information was not, or no longer, available for the other 20 DS individuals whose brains had been acquired many years ago by MBB, TWBB or IOPPN Brain Banks.

**Table 1:** Mean ± SD age at onset, age at death and duration of illness for cases of *APP* duplication (*APP*dup), Down syndrome, missense *APP* mutations and sporadic early onset Alzheimer’s disease (sEOAD), sporadic late onset Alzheimer’s disease and controls, both overall and stratified according to each CAA phenotype

	Age at onset (years)	Age at death (years)	Duration of illness (years)
*APP*dup			
All (*n* = 4)	51.0 ± 5.0^+++^ (44–55)	61.0 ± 5.7^+++^ (55–68)	10.0 ± 3.2 (7–14)
CAA type 2 (*n* = 1)	54.0	68.0	14.0
CAA type 3 (*n* = 3)	50.0 ± 5.6 (44–55)	58.7 ± 4.0 (55–63)	8.7 ± 2.1 (7–11)
Down syndrome^x^			
All (*n* = 34)	53.1 ± 3.7^+++^ (48–59)	58.7 ± 6.0^+++^*** (36–69)	5.9 ± 2.9^!!!^ (2–14)
CAA type 1 (*n* = 15)	52.0 ± 5.0 (50–54)	58.7 ± 8.0 (36–69)	5.0 ± 1.4 (4–6)
CAA type 2 (*n* = 9)	52.6 ± 3.6 (49–57)	58.9 ± 3.2 (53–64)	6.0 ± 1.7 (2–9)
CAA type 3 (*n* = 10)	54.4 ± 6.0 (48–59)	58.4 ± 4.8(47–62)	6.0 ± 4.7(2–14)
Missense *APP*			
All (*n* = 16)	51.6 ± 7.4^+++^ (40–62)	61.8 ± 6.2^+++^ (51–70)	10.8 ± 4.6 (6–21)
CAA type 1 (*n* = 7)	52.0 ± 8.3(42–62)	60.0 ± 6.4 (51–61)	7.8 ± 2.3 (6–12)
CAA type 2 (*n* = 7)	52.3 ± 7.3 (40–59)	64.6 ± 5.6 (59–72)	12.3 ± 4.6 (6–21)
CAA type 4 (*n* = 2)	45.0	58.0 ± 7.7 (53–63)	18.0
sEOAD			
All (*n* = 34)	55.1 ± 5.9^+++^ (35–64)	63.6 ± 5.5^+++^ (45–69)	8.6 ± 2.4 (5–14)
CAA type 1 (*n* = 9)	52.1 ± 7.4(35–60)	62.3 ± 7.3 (45–69)	10.4 ± 1.9** (7–14)
CAA type 2 (*n* = 20)	55.8 ± 4.9 (43–64)	63.7 ± 4.7(50–68)	7.7 ± 2.3 (5–10)
CAA type 3 (*n* = 5)	58.8 ± 2.2 (56–61)	66.6 ± 1.7(64–67)	7.9 ± 1.9(5–10)
sLOAD			
All (*n* = 34)	72.9 ± 7.1 (65–90)	80.8 ± 7.4 (70–95)	8.0 ± 2.7^!^ (3–14)
CAA type 1 (*n* = 17)	73.7 ± 6.8 (65–84)	82.1 ± 7.7 (70–95)	8.4 ± 2.7 (3–14)
CAA type 2 (*n* = 7)	70.1 ± 7.4 (65–86)	77.6 ± 7.2 (71–92)	7.4 ± 3.1 (3–13)
CAA type 3 (*n* = 6)	71.3 ± 6.0 (65–80)	78.8 ± 6.4 (70–87)	8.2 ± 2.6 (4–12)
Controls			
All (*n* = 30)	na	87.0 ± 6.2^+++$$^ (69–100)	na
CAA type 1 (*n* = 15)	na	87.3 ± 6.0 (76–100)	na
CAA type 2 (*n* = 2)	na	87.5 ± 7.1(87–88)	na

Age range is given in parentheses

*na* not applicable

^X^Group age at onset and duration of illness data based on 14 individuals (2 CAA type 1, 7 CAA type 2 and 5 CAA type 3)

**Significantly different from CAA type 2, *p* < 0.01

***Significantly different from sEOAD, *p* < 0.001

^+++^Significantly different from sLOAD group, *p* < 0.001

^$$^Significantly different from sLOAD group, *p* < 0.01

^!,!!!^Significantly different from missense *APP* group, *p* < 0.05, < 0.001, respectively

None of the individuals in any of the pathological groups had been known to have suffered from stroke or ICH.

### Histological methods

Paraffin sections were cut at 6 µm from formalin fixed blocks of frontal lobe (BA8/9), temporal lobe (BA21/22) with posterior hippocampus, occipital lobe (BA17/18) and cerebellar hemisphere. Following titration to determine optimal immunostaining, antibodies were identically employed in a standard immunohistochemical protocol [10]. Sections were immunostained for Aβ using 4G8 antibody (Cambridge Bioscience, clone 4G8, 1:3000) and for tau proteins phosphorylated at Ser202 and Thr205 (P-tau) using AT8 antibody (Source Bioscience, clone AT8, 1:750). Sections immunostained with 4G8 antibody were subject to formic acid pretreatment before antigen unmasking by pressure cooking in citrate buffer (pH 6.0, 10 mM) for 30 min, reaching 120 degrees Celsius and > 15 kPa pressure. The formic acid pretreatment step was omitted for AT8 (tau) immunostaining. Following immunostaining, all cases were staged as Thal phase of Aβ deposition [45] and Braak stage of neurofibrillary degeneration [4]. Additional immunostaining, using previously validated end-specific Aβ_40_ and Aβ_42(3)_ monoclonal antibodies (known as BA27 and BC05, respectively) was performed on 30 cases, selected to be representative of each CAA phenotype within each pathological group, as described previously [17, 18]. For this work, we chose three *APP*dup cases (case #1–3), six with DS (#12, 17, 19–21 and 26), five with missense *APP* mutations (#49–52 and 54), six with sEOAD (#58, 62, 68, 75, 78 and 81), seven with sLOAD (#90, 93, 95–98 and 122) and three controls (#129, 140 and 143).

Sections were examined microscopically for the appearance, severity and topographical distribution of immunostaining within brain parenchyma (as plaques) and cerebral vessels (as CAA). A five-point scoring system, similar to that employed by Olichney et al. [34], was employed to separately grade the severity of Aβ plaques and CAA as total Aβ (4G8) and separately as Aβ_40_ (BA27) and Aβ_42(3)_ (BC05) specific changes.

*Plaques*

- Grade 0—no Aβ plaques in parenchyma.
- Grade 1—A few Aβ plaques in parenchyma occupying each low power (× 10 microscope objective) field.
- Grade 2—A moderate number of Aβ plaques in parenchyma occupying each low power (× 10 microscope objective) field.
- Grade 3—Many dispersed Aβ plaques in parenchyma occupying each low power (× 10 microscope objective) field.
- Grade 4—Very many densely packed Aβ plaques in parenchyma occupying each low power (× 10 microscope objective) field.

*CAA*

- Grade 0—No CAA in blood vessel walls in leptomeninges or brain parenchyma.
- Grade 1—Occasional blood vessels with CAA in leptomeninges and/or within brain parenchyma, usually not occupying the full thickness of the wall.
- Grade 2—A moderate number of blood vessels with CAA in leptomeninges or brain parenchyma in leptomeninges or within brain parenchyma, some occupying the full thickness of the wall.
- Grade 3—Many blood vessels with CAA in leptomeninges or brain parenchyma, most occupying the full thickness of the wall.
- Grade 4—Most or all blood vessels with severe CAA in leptomeninges or within brain parenchyma, occupying the full thickness of the wall.

Representative images for each plaque and CAA grade are presented in Supplementary Fig. 1.

Plaque and CAA scores were separately summated across all four brain regions examined to provide a ‘global’ plaque and CAA score for each patient.

**Fig. 1:**
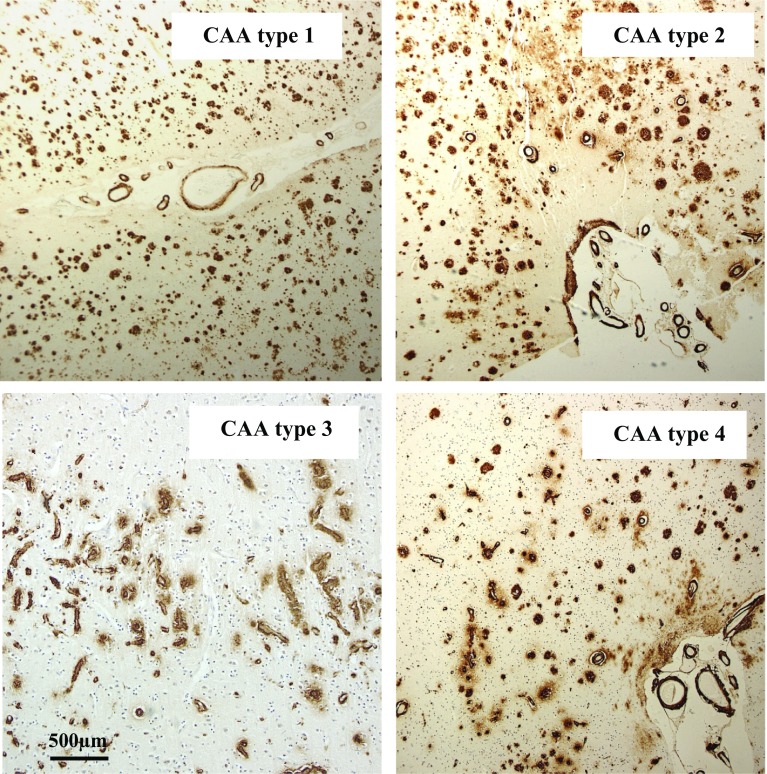
The four different CAA phenotypes as seen on immunostaining for Aβ. Type 1 describes cases predominantly with many diffuse and cored Aβ plaques, throughout the cerebral cortex in which CAA is confined in leptomeningeal vessels. Type 2 describes cases where, along with many diffuse and cored Aβ plaques, CAA is present in both leptomeningeal and deeper penetrating arteries, especially within occipital cortex. Type 3 describes cases where capillary CAA is present along with arterial CAA, especially within primary visual cortex, but with relatively few Aβ plaques. Type 4 describes a predominantly vascular phenotype, where Aβ deposition is much more prevalent in and around blood vessels throughout the brain and Aβ plaques are scarce or absent. Immunoperoxidase–haematoxylin

CAA subtype, based on examination of frontal, temporal and occipital cortex in sections immunostained for Aβ, was assigned to all cases as previously described [2]. Type 1 describes cases predominantly with many diffuse and cored Aβ plaques, throughout the cerebral cortex, in which CAA is confined within leptomeningeal vessels. Type 2 describes cases where, along with many diffuse and cored Aβ plaques, CAA is present in both leptomeningeal and deeper penetrating arteries, especially within occipital cortex. Type 3 describes cases where capillary CAA is present along with arterial CAA, especially within primary visual cortex, but with relatively few Aβ plaques. Type 4 describes a predominantly vascular phenotype, where Aβ deposition is much more prevalent in and around blood vessels throughout the brain and Aβ plaques are scarce or absent. Representative images for each CAA subtype are shown in Fig. 1. All phenotype assessments were performed by a single, highly experienced neuropathologist (Mann), blinded to patient grouping, based on a ‘template’ derived from previous work [2] in which the assessment methodology had been validated.

### APOE genotyping

DNA was extracted from frozen cerebellum or frontal cortex by routine methods from which *APOE* genotype was determined [50]. *APOE* genotyping was possible for all 4 *APP*dup patients, 25/34 DS individuals, 13/16 missense *APP* mutation patients, all 34 sEOAD patients, 33/34 sLOAD patients, and all 30 controls. The lack of available frozen tissue prevented *APOE* genotyping for those remaining individuals.

### Statistical analysis

Rating data were entered into an excel spreadsheet and analysed using Statistical Package for Social Sciences (SPSS) software (version 20.0). Patients were stratified according to genetic subtype for statistical analysis of the effect of each mutation on the underlying Aβ pathology. Comparisons of semi-quantitative scores for intensity of Aβ immunostaining were performed using Kruskal–Wallis test with post hoc Mann–Whitney test where Kruskal–Wallis yielded a significant difference between antibody staining scores. Group comparisons of age at onset, age at death and duration of illness were made using ANOVA with post hoc Tukey test. Comparisons of frequency of *APOE* alleles and genotypes were performed using chi-square test. Correlations between rating data were made using Spearman rank correlation test. In all instances, significance levels were set at *p* < 0.05.

## Results

### Demographic observations

There were significant differences between *APP*dup, DS, missense *APP* mutations, sEOAD and sLOAD groups with respect to mean age at onset of disease (*F*_4,93_ = 52.6, *p* < 0.001). By definition, patients with sLOAD had a significantly later age at onset of illness (*p* < 0.001) compared to those with *APP*dup, DS, missense *APP* mutations and sEOAD, but there were no significant differences in age at onset between any of the early onset AD and DS groups (Table 1). Similarly, there were significant differences between *APP*dup, DS, missense *APP* mutations, sEOAD and sLOAD cases and control groups with respect to mean age at death (*F*_5,146_ = 99.1, *p* < 0.001) with patients with sLOAD, and controls, all (by definition) dying at a later age (*p* < 0.001) than patients with *APP*dup and missense *APP* mutations, sEOAD and individuals with DS. Again, there were no significant differences in age at death between any of the early onset AD and DS groups (Table 1). Patients with sEOAD were slightly older at death than those individuals with DS (*p* = 0.017). The controls died at a later age than the patients with sLOAD (*p* = 0.002) (Table 1). There were also significant differences in mean duration of illness (*F*_4,93_ = 5.1, *p* = 0.001) between patients with *APP*dup, DS, missense *APP* mutations, sEOAD and sLOAD. Patients with missense *APP* mutations had a longer duration of illness than individuals with DS (*p* < 0.001) and patients with sLOAD (*p* = 0.042).

Consistent with general longevity, mean age at onset and mean age at death, were both significantly later in females compared to males in the sLOAD group alone (*p* = 0.004), but no significant gender difference was seen with respect to duration of illness. There were no gender differences in age at onset, age at death or duration of illness for each CAA subtype within each patient group.

### Braak and Thal stageing

Based on AT8 immunostaining for tau proteins (Braak stage) and 4G8 immunostaining for Aβ (Thal phase), all 4 patients with *APP*dup and all 16 with missense *APP* mutations were at Braak tangle stage 6 and Thal amyloid phase 5. Of the 34 individuals with DS, 11 (32%) were at Braak stage 5 and 23 (68%) at Braak stage 6; all were at Thal stage 5. Of the 34 patients with sEOAD, 10 (29%) were at Braak stage 5 and 24 (71%) were at Braak stage 6, and 2 (6%) were at Thal stage 4 and 32 (94%) were at Thal stage 5. Of the 34 patients with sLOAD, 2 (6%) were at Braak stage 4, 6 (18%) were at Braak stage 5 and 26 (76%) were at Braak stage 6, and 6 (18%) were at Thal stage 4 and 28 (82%) were at Thal stage 5. Of the 30 controls, 1 (3%) was Braak stage 0, 13 (43%) were at Braak stage 1 and 16 (54%) were at Braak stage 2, and 9 (30%) were Thal stage 0, 10 (33%) were at Thal stage 1 and 11 (37%) were at Thal stage 2 (see Supplementary Table 1).

### Pathological changes and CAA phenotypes

#### *APP*dup

As evidenced by 4G8 immunostaining, CAA was severe in all four patients, but did not present with a uniform appearance.

**Fig. 2:**
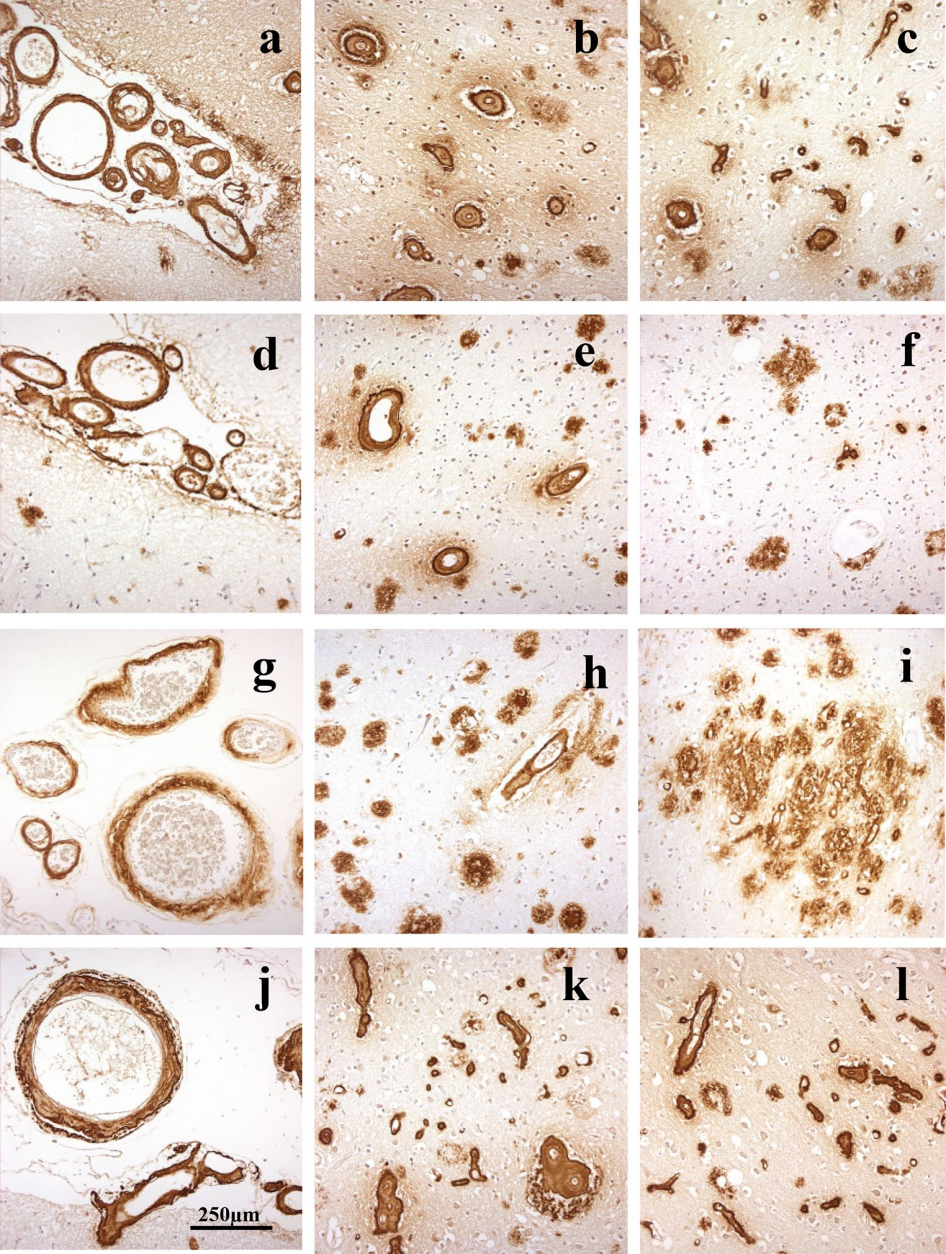
Patterns of immunostaining for Aβ as CAA and plaques in occipital cortex in *APP*dup patients #1 (**a**–**c**), #2 (**d**–**f**), #3 (**g**–**i**) and #4 (**j**–**l**). Immunoperoxidase–haematoxylin

**Fig. 3:**
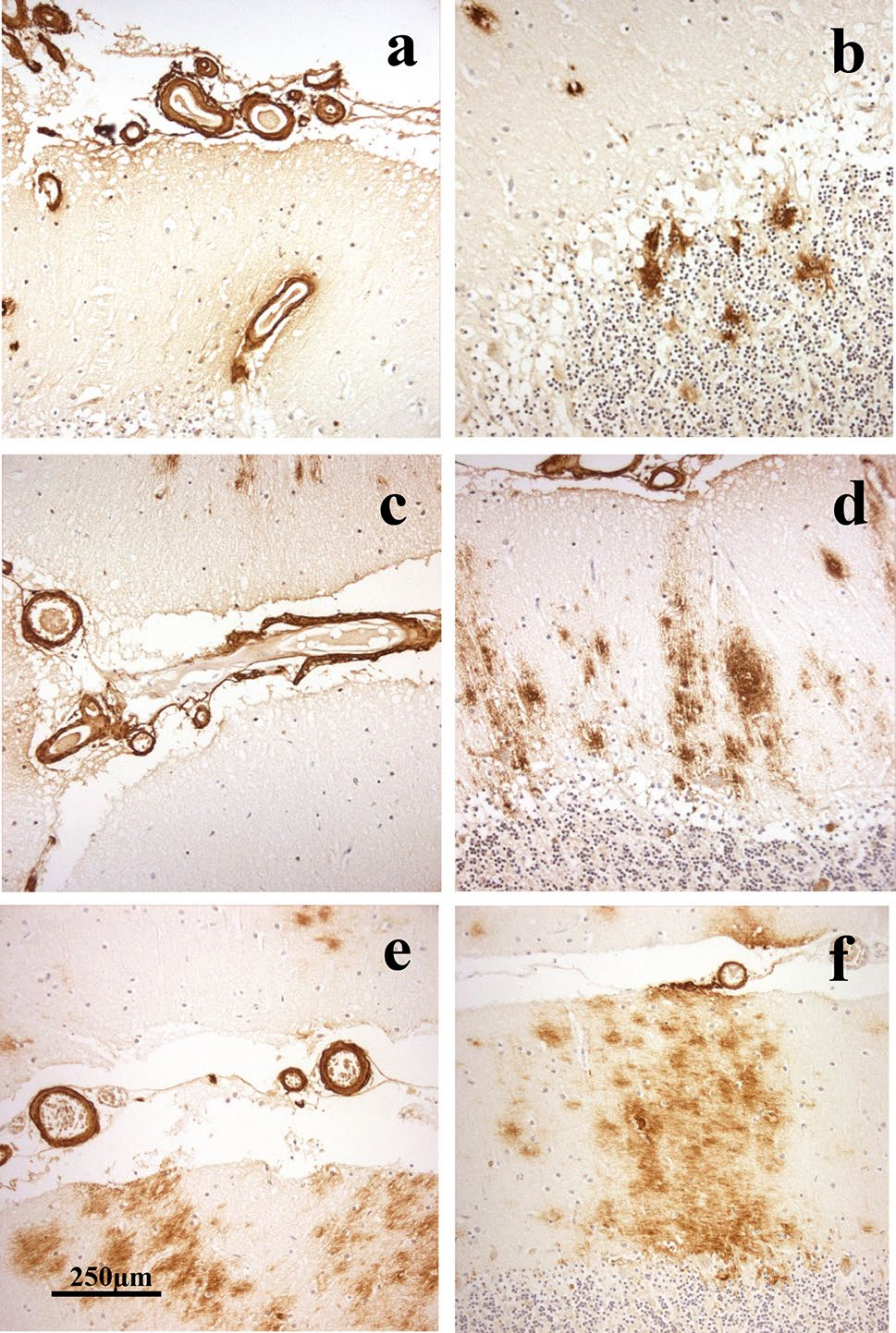
Patterns of immunostaining for Aβ as CAA and plaques in cerebellum in *APP*dup patients #1 (**a**, **b**), #2 (**c**, **d**) and #3 (**e**, **f**). Immunoperoxidase–haematoxylin

In patients #1 (Fig. 2a–c), #3 (Fig. 2g–i) and #4 (Fig. 2j–l), there was very severe involvement of all arteries in occipital cortex, both leptomeningeal and intraparenchymal extending to variable degrees into the capillary bed, with some perivascular deposition of Aβ. There were relatively few amyloid plaques in comparison with the degree of vascular and perivascular involvement, most of these being cored-type plaques (Fig. 2b, h, k). This pattern of CAA, which most closely resembled type 3 (see [2]), was also seen in frontal and temporal cortical regions. CAA was also severe in the cerebellum, but mostly confined to leptomeningeal arteries (Fig. 3a, c, e). There were variable numbers of amyloid plaques, mostly as diffuse deposits within the molecular layer in patient #3 (Fig. 3f), but in patient #1 there were coarser, irregular deposits in Purkinje and granule cell layers (Fig. 3b). The cerebellum was not available for study in patient #4. In general, the severity of the changes in patient #3 was less than that in patients #1 and #4, though they essentially retained the same pathological characteristics.

In patient #2, severe CAA was seen across all three cortical regions affecting leptomeningeal (Fig. 2d) and parenchymal (Fig. 2e) arteries but only very rarely extending into the capillary bed (Fig. 2f). Dense perivascular deposits were not seen, and Aβ plaques were present in all regions. In the cerebellum, moderate CAA was present confined to leptomeningeal arteries (Fig. 3e) but many diffuse deposits were seen in the molecular layer and some coarser deposits in Purkinje cell layer (Fig. 3f). This pattern, in the main, conformed to type 2 CAA [2] though the very limited capillary involvement in occipital cortex (Fig. 2f) would suggest a phenotype verging on type 3 CAA.

#### Down syndrome

As seen in 4G8 immunostaining, amyloid deposition in the form of plaques and CAA was widely present in all 34 individuals with DS. CAA was present as type 1 in 15 (44%) cases, type 2 in 9 (26%) cases and type 3 in 10 cases (29%) (Table 1).

#### Missense *APP* mutations

**Fig. 4:**
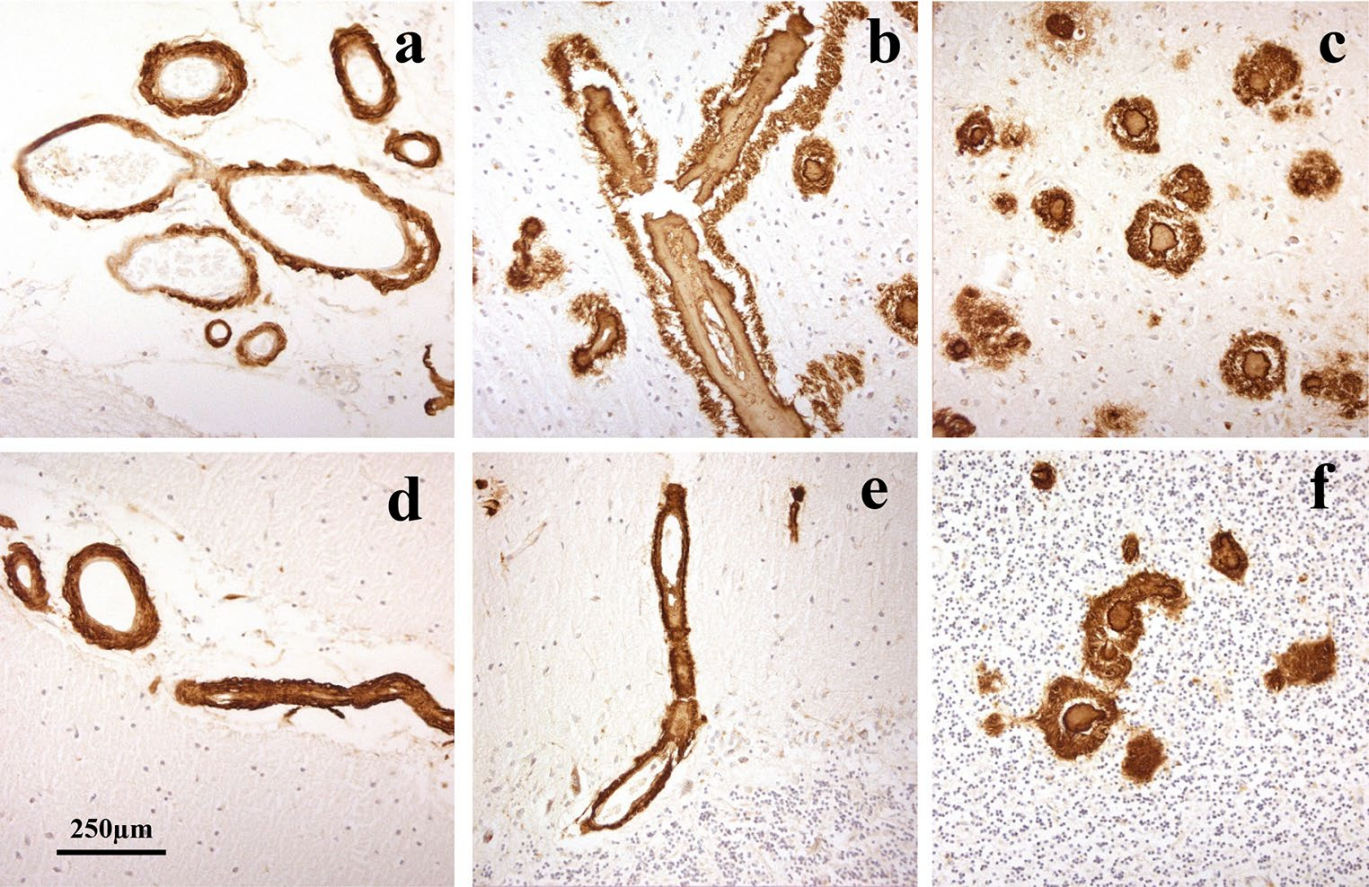
Patterns of immunostaining for Aβ as CAA and plaques in occipital cortex (**a**, **b**) and cerebellum (**c**, **d**) in missense *APP* patient #61. Immunoperoxidase–haematoxylin

By 4G8 immunostaining, CAA was present in all 16 patients with missense *APP* mutations, being type 1 in 7 of the 14 patients with codon 717 mutation and type 2 in the other 7 patients (Table 1). The two patients with *APP*692 mutation displayed a unique phenotype (type 4) in which arteriolar and capillary CAA with perivascular deposition of amyloid (dyshoric angiopathy), but few/no plaques, was predominant within occipital cortex, in addition to severe leptomeningeal CAA (Fig. 4a–c). In the cerebellum, CAA was severe and sometimes affected parenchymal arteries in both the molecular and granule cell layer, again with perivascular deposition especially in granule cell layer (Fig. 4d–f).

#### Sporadic EOAD and LOAD and control subjects

Using 4G8 immunostaining, CAA was present in all 34 patients with sEOAD (as type 1 in 9 patients (26%), type 2 in 20 patients (59%) and type 3 in 5 patients (15%)), in 30/34 patients with sLOAD (as type 1 in 17 patients (57%), type 2 in 7 patients (23%) and type 3 in 6 patients (20%), but only in 17 controls (57%) (as type 1 in 15 subjects (88%) and type 2 in the other 2 subjects (12%) (Table 1).

We carefully examined routine haematoxylin–eosin stained sections from the cortical and cerebellar regions assessed for CAA in all cases for evidence of overt ICH/microbleeds, sections, but found none to be significantly, or at least consistently, present in any patient group or CAA subtype.

### Comparisons of CAA phenotype

**Fig. 5:**
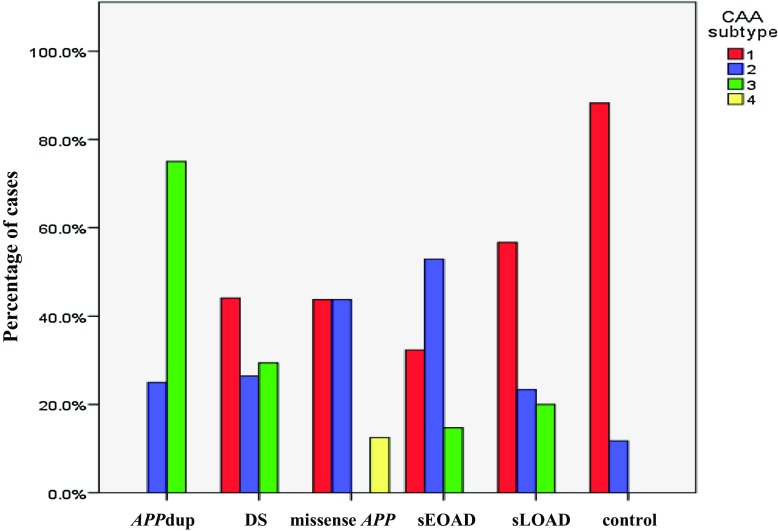
Percentage frequency of the four CAA phenotypes within each of the six different pathological groups

The proportion of patients showing different CAA phenotypes varied between the pathological groups (Fig. 5). The proportion of individuals with DS showing type 1 CAA (44%) was significantly greater (*p* = 0.003) than those with sEOAD (27%) but not those with sLOAD (57%). Also, the proportion of individuals with sLOAD (57%), and that of controls (88%), showing type 1 CAA was both significantly greater than that proportion of sEOAD patients with type 1 CAA (*p* = 0.014 and 0.0003, respectively). Conversely, the proportion of patients with sEOAD with type 2 CAA (59%) was significantly greater than that in those with DS (24%, *p* = 0.002) or sLOAD (23%, *p* = 0.004) or in controls (12%, *p* = 0.001). For type 3 CAA, the proportion of individuals with *APP*dup (75%) was significantly greater than those with DS (27%, *p* = 0.014), sEOAD (15%, *p* = 0.005) and sLOAD (20%, *p* = 0.019), and was also greater (*p* < 0.05 by Fisher exact test) than those with missense *APP* mutations and controls (0% in both).

When stratified according to each CAA phenotype, there were no significant differences with respect to mean age at onset of disease for patients with *APP*dup, missense *APP* mutations, sEOAD and sLOAD, or for mean age at death between any of the CAA phenotypes for *APP*dup, DS, missense *APP* mutations, sEOAD, sLOAD and control cases (Table 1). Only in sEOAD group did duration of illness vary (*F*_2,29_ = 5.5, *p* = 0.01) between CAA phenotypes, with CAA type 1 cases having a significantly longer duration of illness than those with type 2 CAA (*p* = 0.01) (Table 1).

### Comparisons of plaque and CAA scores

**Fig. 6:**
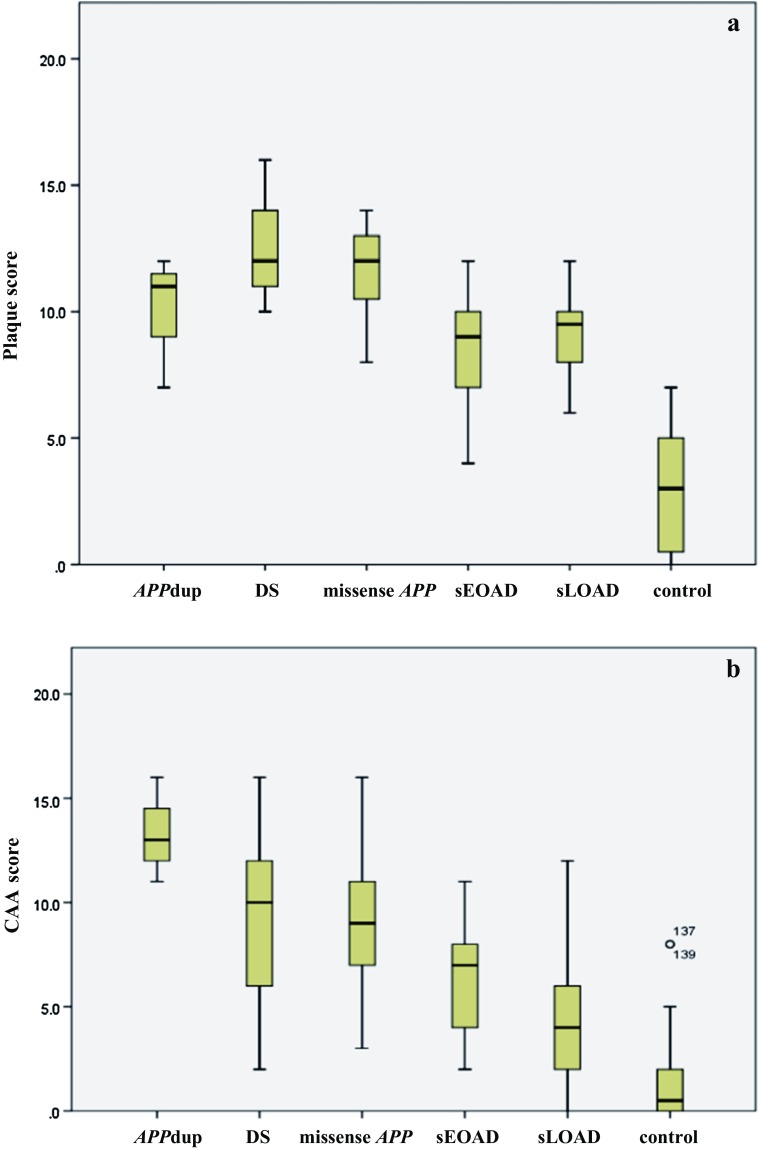
Box plots of scores for severity of plaques (**a**) and CAA (**b**) across the six different pathological groups. ^+++^Significantly different (*p* < 0.001) from controls. ***Significantly different (*p* < 0.001) from both sEOAD and sLOAD. ^!^Significantly different (*p* < 0.05) from DS. ^$^Significantly different (*p* < 0.05) from sLOAD

Both overall plaque (*χ*^2^ = 109.2, *p* < 0.001) (Fig. 6a) and CAA (*χ*_2_ = 68.0, *p* < 0.001) scores differed significantly across the six groups. The degree of plaque formation (overall plaques scores) was significantly greater in both DS and missense *APP* mutations than in sEOAD and sLOAD cases (*p* < 0.001 in every instance). As would be expected, all 5 pathological (AD) groups had a significantly greater degree of plaque formation than controls (*p* < 0.001), but the degree of plaque formation was not significantly different between sEOAD and sLOAD cases (Fig. 6a).

As would be expected, all five pathological (AD) groups had a significantly greater degree of CAA than controls (*p* < 0.001) but, in contrast to plaque scores, the degree of CAA was also significantly greater in sEOAD than sLOAD (*p* = 0.014) (Fig. 6b). On the other hand, the severity of CAA was significantly greater in both *APP*dup (*p* = 0.005 and *p* = 0.009, respectively), missense *APP* mutations (*p* = 0.016 and *p* = 0.001, respectively) and DS (*p* = 0.008 and *p* < 0.001, respectively) than in sEOAD and sLOAD, and the severity of CAA was greater in *APP*dup than that in DS (*p* = 0.014) but not so for missense *APP* mutations (*p* = 0.056), with there being no significance difference between the missense *APP* mutations and DS (Fig. 6b).

There were no significant differences between males and females in either plaque or CAA scores for each pathological group, or for each CAA subtype within each pathological group.

### Relationship between CAA subtypes and plaque or CAA severity

When stratified by CAA subtype, there were no significant differences in overall plaque scores between each CAA subtype for any of the six groups. However, overall severity of CAA did vary significantly across subtypes for DS (*χ*^2^ = 13.0, *p* < 0.001), sEOAD (*χ*^2^ = 13.1, *p* = 0.001) and sLOAD (*χ*^2^ = 14.4, *p* < 0.001), but not for patients with *APP*dup or missense *APP* mutation, or controls. In both missense *APP* mutations and sEOAD there was a significantly greater level of CAA as both CAA type 2 (*p* = 0.001 and 0.004, respectively) and type 3 (*p* < 0.001 and 0.002, respectively) than as type 1, with no significant differences in CAA severity between type 2 and type 3 cases. In DS, sLOAD and controls, there was a significantly greater level of CAA in type 1 than type 2 and type 3 (*p* < 0.006 and 0.002), with no significant differences in CAA severity between type 2 and type 3 cases.

**Fig. 7:**
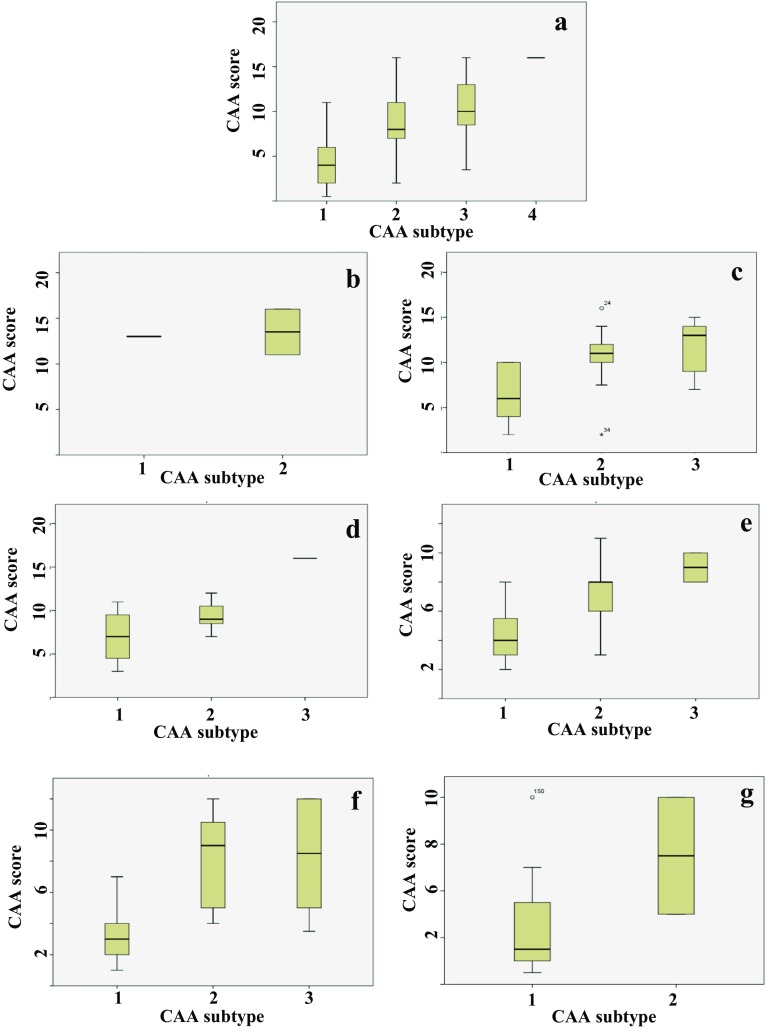
Box plots of scores for CAA severity scores across the four different CAA phenotypes for all cases collectively (**a**) and individually for APPdup (**b**), DS (**c**), missense *APP* mutations (**d**), sEOAD (**e**), sLOAD (**f**) and control (**g**) groups

There were significant correlations between CAA severity scores and CAA phenotype when all cases were considered together (*r*_s_ = 0.646, *p* < 0.001) or separately as individual pathological groups (DS, *r*_s_ = 0.604, p < 0.001; missense *APP* mutations, *r*_s_ = 0.612, *p* = 0.012; sEOAD, *r*_s_ = 0.628, *p* < 0.001; sLOAD, *r*_s_ = 0.680, *p* < 0.001). For controls, this correlation failed to reach significance (*r*_s_ = 0.377, *p* = 0.136). The number of cases with *APP*dup was too low to permit this particular analysis. For all groups, CAA severity scores progressively increased with ascending CAA phenotype class (Fig. 7).

### Comparisons of patterns of immunostaining using end-specific antibodies

**Fig. 8:**
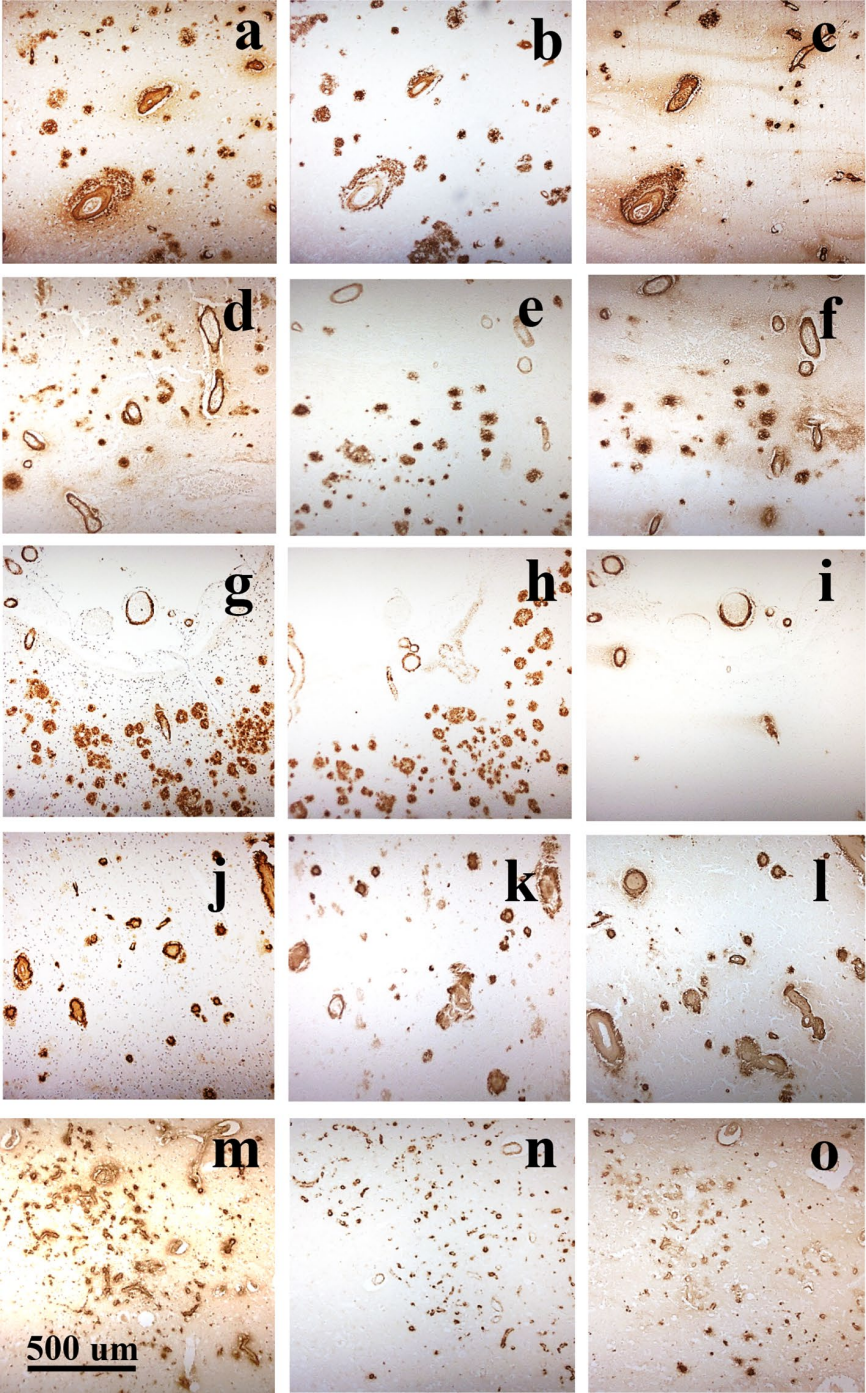
Adjacent sections of occipital cortex from *APP*dup case #1 (**a**–**c**), DS case #17 (**d**–**f**), missense *APP* mutation cases #49 (**g**–**i**) and #54 (**j**–**l**) and sEOAD case #58 (**m**–**o**) immunostained for Aβ using 4G8 antibody to detect total Aβ (**a**, **d**, **g**, **j**, **m**), BC05 antibody to detect Aβ_42(3)_ (**b**, **e**, **h**, **k**, **n**) and BA27 antibody to detect Aβ_40_ (**c**, **e**, **i**, **l**, **o**). All blood vessels stained for CAA by 4G8, irrespective of genetic or pathological group, or CAA phenotype, also appeared to be strongly immunoreactive for Aβ_40_ but less strongly for Aβ_42(3)_. On the other hand, all plaques detected by 4G8 were strongly immunoreactive for Aβ_42(3)_ but only a subset (of cored plaques) appeared to contain Aβ_40._ Immunoperoxidase (with haematoxylin counterstain in **a**, **d**, **g**, **j**, **m**)

The pattern of immunostaining for Aβ as plaques or CAA was compared for 4G8, BC05 and BA27 antibodies. Irrespective of pathological/genetic grouping, all blood vessels stained for CAA by 4G8 also appeared to be detected by BA27 but fewer were detected (and less strongly so) with BC05. On the other hand, all plaques detected by 4G8 also appeared to be detected by BC05, but only a subset (of cored plaques) was detected by BA27 (Fig. 8).

Semi-quantitative analysis of the rating data for the level of immunostaining of plaques and CAA by 4G8, BC05 and BA27 antibodies was performed on the 30 cases collectively. The degree of immunostaining of plaques differed significantly between the three antibodies (*χ*^2^ = 37.9, *p* < 0.001) with scores for rating of plaque density with 4G8 antibody being significantly greater than that for BC05 antibody (*p* = 0.019) and BA27 antibody (*p* < 0.001), and that for BC05 also being significantly greater than BA27 (*p* < 0.001). Similarly, the degree of immunostaining for CAA differed significantly between the three antibodies (*χ*^2^ = 6.8, *p* = 0.033) with scores for rating of CAA density with 4G8 and BA27 antibodies being significantly greater than that for BC05 antibody (*p* = 0.035 and 0.019, respectively). No significant difference between scores for 4G8 and BA27 antibodies was found (*p* = 0.582). Despite there being significant differences in the levels of immunostaining for plaques and CAA between the three antibodies, regression analysis showed highly significant correlations between plaque scores for each of the three antibodies (*p* < 0.001 in every instance), and likewise for CAA scores (*p* < 0.001 in every instance).

### Effect of APOE genotype

Overall, *APOE* ε4 allele frequency in sEOAD patients was significantly higher than that in DS individuals (*p* < 0.0001), missense *APP* patients (*p* < 0.001) and controls (*p* < 0.001), as was this in sLOAD patients compared to DS (*p* < 0.001), missense *APP* mutations (*p* < 0.001) and controls (*p* < 0.001) (Table 2). There were no significant differences in ε4 allele frequency between DS and missense *APP* patients, between DS and controls, or between sEOAD and sLOAD patients (Table 2). None of the *APP*dup patients bore *APOE* ε4 allele and, therefore, could not be included in the comparisons.

**Table 2:** *APOE* allele and genotype numbers (percentage frequency in parentheses) in *APP* duplication (*APP*dup), Down syndrome, missense *APP* mutations, sporadic early onset Alzheimer’s disease (sEOAD), late onset Alzheimer’s disease (sLOAD) and controls

	*APP*dup (*n* = 4)	Down syndrome (*n* = 25)	Missense *APP* (*n* = 13)	sEOAD (*n* = 34)	sLOAD (*n* = 33)	Controls (*n* = 30)
ε2/ε2	0 (0)	0 (0)	0 (0)	1 (2.1)	0 (0)	0 (0)
ε2/ε3	0 (0)	5 (17.8)	0 (0)	2 (4.2)	1 (3.0)	5 (16.7)
ε2/ε4	0 (0)	1 (3.6)	0 (0)	0 (0)	0 (0)	1 (3.3)
ε3/ε3	4 (100)	16 (57.1)	11 (84.6)	14 (41.2)	7 (21.2)	20 (66.7)
ε3/ε4	0 (0)	6 (21.4)	2 (15.4)	9 (26.5)	16 (48.4)	4 (13.3)
ε4/ε4	0 (0)	0 (0)	0 (0)	8 (23.5)	9 (27.3)	0 (0)
ε2	0 (0)	6 (10.7)	0 (0)	4 (6.0)	1 (1.5)	6 (10.0)^+^
ε3	8 (100)	44 (78.6)	24 (92.3)	39 (57.3)	31 (46.9)	49 (81.2)
ε4	0 (0)	6 (10.7)^***,+++^	2 (7.7)^***,+++^	25 (36.7)	34 (51.5)	5 (8.8)^***,+++^

***Significantly different from sEOAD, *p* < 0.001

^+,+++^Significantly different from sLOAD, *p* < 0.05, < 0.001 respectively

There were no significant differences in *APOE* ε2 allele frequency between any of the groups, except that this was significantly higher in controls (*p* = 0.038) compared to sLOAD patients (Table 2). None of the *APP*dup or missense *APP* mutation patients bore *APOE* ε2 allele (Table 2) and so could not be included in these comparisons.

There was no significant effect of possession of at least one copy of *APOE* ε4 allele, or possession of none, one or two ε4 alleles, on overall plaque severity in DS, sEOAD or sLOAD. Likewise, there was no significant effect of possession of at least one copy of *APOE* ε4 allele on overall CAA severity in DS and sEOAD, but in sLOAD severity of CAA was significantly greater in *APOE* ε4 allele bearers compared to non-bearers (*p* = 0.040). Moreover, when stratified according to possession of none, one or two ε4 alleles, the severity of CAA in sLOAD was significantly greater in patients with two *APOE* ε4 alleles than both those with one or no *APOE* ε4 alleles (*p* = 0.013 in both instances) with no significant difference between those with one or no *APOE* ε4 alleles. No such effect was seen in sEOAD. It was not possible to test for the effects of homozygosity for *APOE* ε4 allele in DS as there were no such individuals represented in the cohort. Likewise, the effects of possession of *APOE* ε4 allele could not be tested in *APP*dup or missense *APP* mutation due to lack of patient representation.

Due to the low frequency of *APOE* ε4 allele, it was not possible to ascertain whether *APOE* genotype influenced CAA phenotype in patients with *APP*dup and missense *APP* mutations (Tables 2, 3). Comparisons of *APOE* ε4 allele frequency between each of the CAA phenotypes within DS, sEOAD, sLOAD and control groups showed no significant differences, except that *APOE* ε4 allele frequency was significantly greater in type 1 CAA and type 3 CAA in comparisons between sEOAD and sLOAD, and sEOAD and controls (Table 3). By contrast, the proportion of ε4ε4 homozygotes in sEOAD and sLOAD groups combined was numerically greater in type 2 (32%) and type 3 (67%) CAA compared to type 1 (11%), and the proportion of these was significantly greater in type 3 CAA (*p* = 0.004), and tending to be greater in type 2 CAA (*p* = 0.065), compared to type 1 CAA, with no significance between type 2 and type 3 CAA.

**Table 3:** Number (percentage in parentheses) of cases in *APP* duplication (*APP*dup), Down syndrome, missense *APP* mutations, sporadic early onset Alzheimer’s disease (sEOAD), sporadic late onset Alzheimer’s disease (sLOAD) and controls with each CAA phenotype (upper half)

	*APOE* ε4 allele frequencies in each CAA phenotype			
	1	2	3	4
*APP*dup (*n* = 4)	0.000	0.000	0.000	0.000
Downs syndrome (*n* = 28)	0.100	0.188	0.100	0.000
Missense *APP* (*n* = 13)	0.000	0.071	0.000	0.250
sEOAD (*n* = 34)	0.272**	0.388	0.500	0.000
sLOAD (*n* = 30)	0.438***	0.643	0.833	0.000
Controls (*n* = 17)	0.067	0.25	0.000	0.000

Also shown (lower half) are *APOE* ε4 allele frequencies associated with each CAA phenotype

**,***Significantly different from controls, *p* < 0.01, < 0.001, respectively

## Discussion

In the present study, we have compared the extent and pattern of Aβ deposition, both as plaques and as CAA, in patients with duplications in *APP*, others with missense mutations in *APP*, patients with sEOAD and sLOAD, older individuals with DS and elderly controls. The main findings to emerge were:

1. The degree of plaque formation was greater in both DS and missense *APP* mutations than in sEOAD and sLOAD cases, while the degree of plaque formation was not significantly different between sEOAD and sLOAD.
2. Conversely, the severity of CAA was significantly greater in both *APP*dup and missense *APP* mutations, and DS, compared to sEOAD and sLOAD. Again, all five pathological (AD) groups had a significantly greater degree of CAA than controls, and the degree of CAA was also significantly greater in sEOAD than sLOAD, and in *APP*dup compared to DS.
3. When stratified by CAA subtype, no significant differences in overall plaque scores were seen between each CAA subtype for any of the six groups. However, in both *APP* mutations and sEOAD there was a significantly greater level of CAA as types 2 and 3 CAA compared to type 1. Conversely, in DS, sLOAD and controls there was a significantly greater level of CAA in type 1 CAA than types 2 and 3. In *APP*dup type 3 was the predominant CAA phenotype.
4. CAA severity scores progressively increased across CAA types 1–4 for all cases combined and for each pathological group individually.
5. *APOE* ε4 allele frequency was overall significantly higher in sEOAD than in DS, missense *APP* and controls, as was ε4 allele frequency in sLOAD compared to DS, missense *APP* mutations and controls. There were no significant differences in *APOE* ε4 allele frequency between DS, missense *APP* and controls. None of the *APP*dup patients bore *APOE* ε4 allele.
6. All blood vessels stained for CAA by 4G8 appeared to be detected by BA27 but fewer were detected with BC05. Conversely, all plaques detected by 4G8 appeared to be detected by BC05, but only a subset was detected by BA27.
7. *APOE* ε4 allele frequency varied numerically between each of the CAA phenotypes in *APP*dup, missense *APP* mutations, sEOAD, sLOAD, DS and controls, but not significantly so. Nonetheless, *APOE* ε4 homozygosity was more commonly associated with type 3 CAA than types 1 and 2 CAA, and was associated with a greater severe of CAA overall in sLOAD but not in sEOAD or DS.

In a recent study, Head and colleagues [15] compared the overall extent of CAA, atherosclerosis and arteriolosclerosis in 32 individuals with DS, ranging in age from 43 to 70 years, and in 80 individuals mostly with late onset sporadic AD (sLOAD) and 37 controls. Younger patients with sEOAD and *APP* mutations were not specifically investigated in this latter study. Nonetheless, like ourselves, these authors found that CAA occurred at significantly higher frequencies in the brains of individuals with DS compared to sLOAD cases and controls, with the DS cohort being 1.2 times more likely to have CAA relative to sLOAD cases, and 4.6 times more likely to have CAA compared to control cases. On the other hand, atherosclerosis and arteriolosclerosis were rare in cases with DS. Such observations of significantly more frequent CAA, and a greater severity of CAA when present, in people with DS relative to sLOAD and control cases are consistent with the hypothesis that such changes are driven, at least partially, by an overexpression of *APP*. The lack of AD neuropathology and CAA, even at greater than 70 years of age, in rare cases of DS with partial trisomy 21 where *APP* is not overexpressed [12, 35] would be consistent with this argument.

Nonetheless, how an overexpression of *APP* might be translated into enhanced CAA remains unclear, but could involve deficiencies in clearance mechanisms when faced with such an overload of Aβ. In this latter context, it has been postulated that the strong association between age, CAA and AD pathology in the general population is driven, at least partially, by an impaired efficiency of cerebral vessels in later life in expelling extracellular fluid containing soluble forms of Aβ as a consequence of atherosclerosis/arteriosclerosis within such vessels [49]. Nonetheless, individuals with DS appear to be protected against hypertension [1] and atherosclerosis, and show less cerebrovascular pathology typically associated with cardiovascular risk factors, including atherosclerotic lesions and arteriolosclerosis [15, 22]. Paradoxically, this ought to result in a better preservation of perivascular drainage channels in DS, and consequently a less severe, rather than more severe, CAA compared to sLOAD. Potential inefficiencies in perivascular drainage might not appertain to individuals with *APP* mutations or DS since these would only be anticipated to occur beyond an age at which most would generally survive to. However, Aβ can also be cleared from the brain through several other routes, involving endocytosis by microglia and astrocytes, or enzymatic degradation. It is, therefore, possible that failures in these latter pathways, in conjunction with the overexpression of APP, might foster an inability to expel Aβ and result in severe CAA.

Despite the commonality of sharing duplication at the *APP* locus, and an overexpression of APP protein, there are clear differences in clinical presentation between *APP*dup and DS individuals. Stroke and ICH are the main clinical consequences of vascular amyloidosis in *APP*dup, occurring in at least one-third of all cases, but both are uncommon in individuals with DS, with only a handful of case reports of this in the literature (see [5]). Indeed, it has been estimated that haemorrhagic stroke occurs in only about 3–4% of older people with DS [43], some ten times less frequent than in patients with *APP*dup. Why these clinical differences should occur is unclear, given that DS differs from *APP*dup only in the number of other genes located on chromosome 21 that are also triplicated. In most instances of DS, a full triplication of chromosome 21 is present, whereas in *APP*dup triplication of *APP* locus is variable, generally ranging from 0.5 to 6.5 Mb [48], with the region of triplication in some instances being limited to *APP* gene alone [41], while in others it may extend to include up to 12 other genes [37]. This raises the question as to whether the possession of other triplicated genes in some way confer some degree of protection against the likelihood of stroke or ICH in DS. These genes may be involved in the production of Aβ, of which there are two main pathways—the secretory pathway or the endo-lysosomal pathway. In the latter, Aβ cleavage occurs in the endosomal compartment where pH is optimal for β-secretase activity. Enlargement of the early endosomal compartment is considered one of the earliest morphological alterations detectable in postmortem tissues in sporadic AD, in most *APP* mutations and in DS [8]. *APP* overexpression is implicated in the formation of enlarged endosomes, but the mechanism is uncertain. Interestingly, it was recently shown that increase of β-CTF, the C-terminal fragments of APP generated after β-secretase cleavage, can produce enlarged endosomes in fibroblasts from DS individuals [20] while in lymphoblastoid cell lines from individuals with *APP*dup endosomes appeared to be of normal size [9].

However, with regards to clinical phenotype, it is notable that in the Dutch family [41] where triplication of *APP* locus was restricted to *APP* gene alone, and in the Swedish family the region of triplication extended to cover only those two genes either side of *APP*, no instances of ICH were reported [48]. Conversely, ICH commonly occurred in a Finnish family with 0.55 Mb duplication covering *APP* and four other neighbouring genes [38]. By contrast, in the French families where there is a greater and more variable extension of triplication of *APP* locus [6, 37], ICH occurred in about 30% of patients though, notably, dementia but not ICH defined the clinical course of the four *APP*dup patients studied here in whom the region of triplication was 0.78 Mb [6]. Hence, factors other than size of *APP* triplication per se may account for the low prevalence of ICH in DS compared to *APP*dup.

Alternatively, it may be differences in the actual CAA phenotype present that are responsible. Type 1 CAA tended to be more common in DS and sLOAD, and type 2 CAA was more common in missense *APP* mutations and sEOAD than in the other groups. Type 3 CAA was more common in individuals with *APP*dup than those with DS as well as others with sEOAD or missense *APP* mutations. Type 4 was only seen in patients with *APP*692 mutation, though 2 of the *APP*dup patients (patients #1 and 3), although designated as CAA type 3, showed a particularly severe phenotype that was reminiscent of type 4 CAA. Consequently, patients with *APP*dup in the present study had a more severe CAA phenotype than most individuals with DS, despite the similar ages, with only 30% DS individuals showing this type 3 phenotype, and then not to the same degree of severity as in patients with *APP*dup. It is possible, therefore, that it is the lesser extent of CAA in DS (compared to *APP*dup) that lowers the risk of CAA-related stroke and haemorrhage in such individuals. Correlations between CAA severity scores and CAA phenotype suggest that the different CAA phenotypes exist on a continuum with type 1 being the least ‘aggressive’ form, and types 2–4 following progressively as Aβ becomes deposited further along the arterial tree reaching into parenchymal arteries and arterioles (type 2) and finally into capillaries (types 3 and 4). Certainly, this would accord with the proposal put forward by Weller et al. [49] based on a progressive slowing of perivascular drainage leading to build up of deposits in vessel walls. However, if so, this would not explain the relative absence of amyloid plaques in types 3 and 4 CAA, and the highest levels of plaques in type 1 CAA.

APP is processed by proteolytic enzymes known as secretases. Cleavage within the APP domain containing the Aβ sequence by α-secretase precludes its formation, whereas sequential cleavage at the amino- and carboxyl- termini of the Aβ sequence by β- and γ-secretases, respectively, releases Aβ into the extracellular fluid of the brain. Most of this occurs as a more slowly aggregating form, Aβ_40_, compared to the longer, more rapidly aggregating form, Aβ_42(3)_. It has been shown that in both AD and DS the Aβ in CAA is composed mostly of Aβ_40_, whereas in plaques it is Aβ_42(3)_ that predominates [17, 18, 40, 44]. The differential pattern of composition of Aβ within brain parenchyma and blood vessel walls can be explained by the relative aggregation properties of Aβ_40_ and Aβ_42(3),_ with the less aggregation prone Aβ_40_ travelling further along perivascular drainage channels and ultimately reaching blood vessel walls, compared to the less abundant though more rapidly aggregating Aβ_42(3)_ which coalesces into plaques within the brain parenchyma. In familial AD due to certain missense mutations in *APP* (for example those at or around codon 717) proteolytic processing of APP elevates levels of Aβ_42(3)_ relative to Aβ_40_ [39] with excessive numbers of Aβ_42(3)_ containing plaques being formed as a consequence [26]. Other missense mutations (for example, those around codons 692 and 693) enhance the aggregation properties of both Aβ_40_ and Aβ_42(3)_ without affecting levels of production. As mentioned earlier triplication at *APP* locus will increase production of both Aβ_40_ and Aβ_42(3)_.

Hence, the different CAA phenotypes seen here in the different forms of AD might in some way reflect the relative proportions of Aβ_40_ and Aβ_42(3)_ being generated. In *APP*dup and DS, where excess amounts of Aβ_40_ (and Aβ_42(3)_) are produced, this could lead to failure to expel this from the extracellular fluid leading to a massive build up in smaller arteries and capillaries evidenced as the more extensive type 3 CAA. In missense *APP* mutations such as those at codon 692, the mutated, more highly aggregation prone, form of Aβ_40_ would promote its excessive deposition in vessel walls, and again result in the severe type 4 CAA (see [26] for *APP*693 mutations), whereas no excess of Aβ_40_ is generated in missense mutations in *APP* occurring around codon 717, and in these cases the less extensive type 1 and 2 CAA predominate. In sEOAD and sLOAD, where normal levels of both Aβ_40_ and Aβ_42(3)_ are produced, the different CAA phenotypes present might reflect the relative efficiencies in which Aβ_40_ is cleared through the perivascular drainage channels. Present observations that all blood vessels stained for CAA by 4G8, irrespective of genetic or pathological group, or CAA phenotype, also appeared to be strongly immunoreactive for Aβ_40_ but less strongly for Aβ_42(3)_. On the other hand, all plaques detected by 4G8 were strongly immunoreactive for Aβ_42(3)_ but only a subset (of cored plaques) appeared to contain Aβ_40_. Such findings are consistent with our previous studies in familial AD and DS [17, 18]. Consequently, in addition to potential differences in Aβ production, differences in CAA phenotypes between DS and *APP*dup might also involve amyloid clearance, but alternative mechanisms could involve a unique oxidative stress profile or immune response in DS [52].

Interestingly, as others have shown [34, 40, 46], possession of two copies of *APOE* ε4 allele was associated with a greater severity of CAA in sLOAD patients alone. However, *APOE* genotype per se did not greatly influence the actual CAA phenotype in any pathological group. Although there was a numerical increase in ε4 allele frequency from type 1 to type 3 CAA in sEOAD and sLOAD, these differences were not substantiated statistically. Nonetheless, it is known that an increase in ε4 allele copy from 0 to 2 is associated with higher levels of Aβ_40_ deposition (as plaques) in sLOAD [13, 27], possession of ε4 allele/ApoE E4 isoform decreases brain clearance of Aβ [7], and ApoE E4 isoform promotes fibrillogenesis [23]. In these respects, possession of *APOE* ε4 allele/E4 isoform could potentiate the development of CAA, as type 3 CAA, in those patients with sLOAD bearing *APOE* ε4ε4 genotype, a suggestion in keeping with previous studies [46]. The absence of type 3 CAA in elderly controls and missense *APP* mutations involving codon 717, would also accord with the relative infrequency of *APOE* ε4 allele and ε4ε4 homozygosity in such individuals. However, having said that, overall severity of CAA and prevalence of type 3 CAA were equivalent in DS individuals as in patients with sEOAD and sLOAD, despite there being in DS a relative lack of *APOE* ε4 alleles, and a complete absence of ε4ε4 homozygotes.

While there were no significant differences in overall plaque scores between sEOAD and sLOAD cases, overall CAA scores were lower in sLOAD than sEOAD. Furthermore, age at death did not vary significantly between CAA phenotypes for these two groups though the proportion of cases with the less severe type 1 CAA was greater in sLOAD than sEOAD, suggesting that advancing age per se may, if anything, lessen the severity of CAA and phenotype present, at least in AD. This observation was not due simply to a shorter duration of disease in sLOAD compared to sEOAD which might potentially have terminated disease progression at an early stage in the later onset cases.

Hence, the factors that determine CAA phenotype are complex and remain unclear, possibly involving differential levels of production or clearance of Aβ_40_ or shorter sized peptides, or factors which promote its aggregation such as ApoE E4 isoform, or some combination of all of these. Moreover, why there should be a distinction in CAA phenotype profiles between DS and *APP*dup is puzzling and it is curious why sEOAD should be more strongly associated with type 2 CAA compared to sLOAD (and vice versa for type 1 CAA), but differential possession of *APOE* ε4 allele does not appear to determine this. Interestingly, previous studies [28] have shown type 2 CAA to be particularly common in early onset familial AD associated with *PSEN*-*1* mutations, especially in those where the mutation is located after codon 200, in the absence of any *APOE* ε4 allele modifying effect. Possibly sEOAD shares some genetic or mechanistic risk affinity with such *PSEN*-*1* mutations, which ultimately translate into a similar CAA phenotype. The neuropathological differences between the different forms of AD highlighted in this study require further study to elucidate the underlying mechanisms. The scientific value in knowing what CAA phenotype is present will help to reduce variability of findings, and provide greater consistency of results, when factors relating to promotion of CAA are being investigated.

## Electronic supplementary material

Below is the link to the electronic supplementary material.

Supplementary material 1 (DOCX 4649 kb) Supplementary Fig. 1. Representative examples of the different plaque and CAA scores. Panel a, plaque score = 0, CAA score = 2. Panel b, plaque score = 1, CAA score = 0. Panel c, plaque score = 2, CAA score = 3. Panel d, plaque score = 3, CAA score = 4. Panel e, plaque score = 3, CAA score = 1. Panel f, plaque score = 4, CAA score = 2. Immunoperoxidase-haematoxylin

Supplementary material 2 (XLSX 19 kb) Supplementary Table 1. Clinical, pathological and genetic characteristics of the patient and control study groups
