# Supplementary figures and images for: Patterns and severity of vascular amyloid in Alzheimer’s disease associated with duplications and missense mutations in APP gene, Down syndrome and sporadic Alzheimer’s disease
Source: Acta Neuropathol. 2018 May 16;136(4):569–87. doi: 10.1007/s00401-018-1866-3 (PMC6132946; doi:10.1007/s00401-018-1866-3)

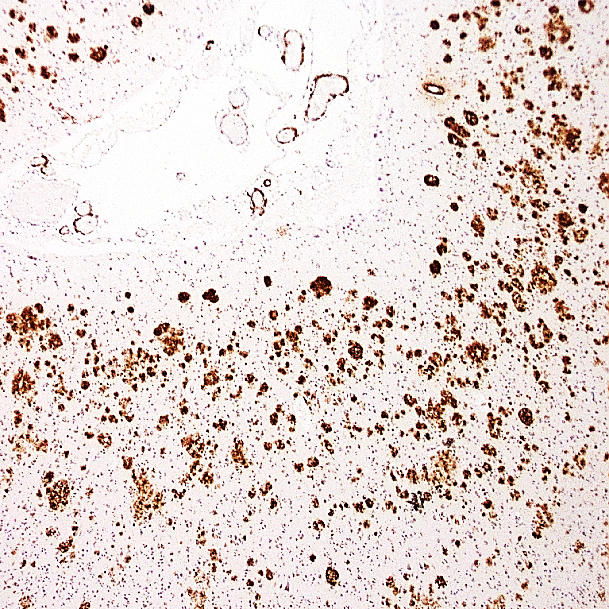

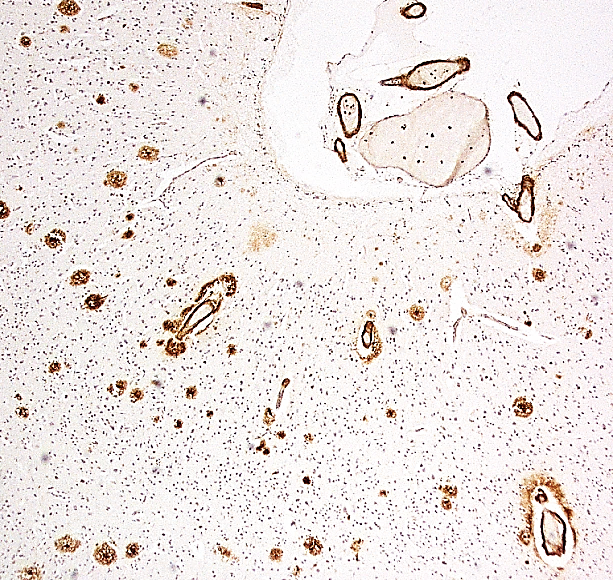

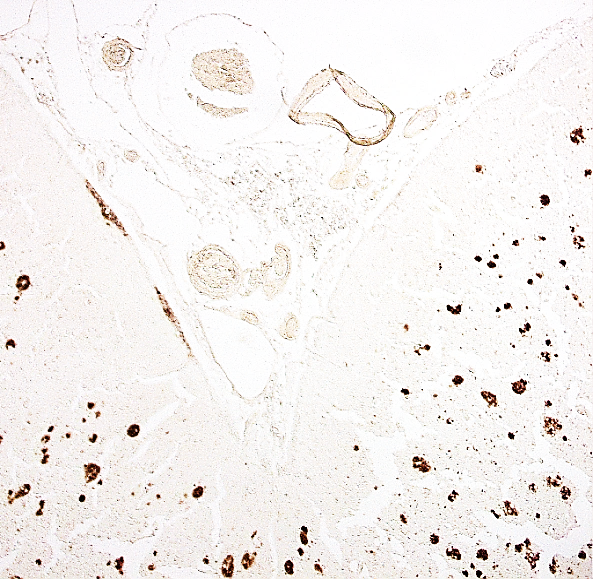

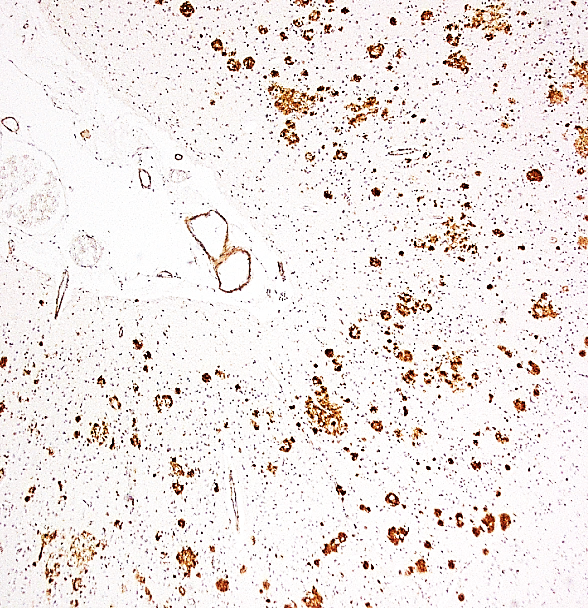

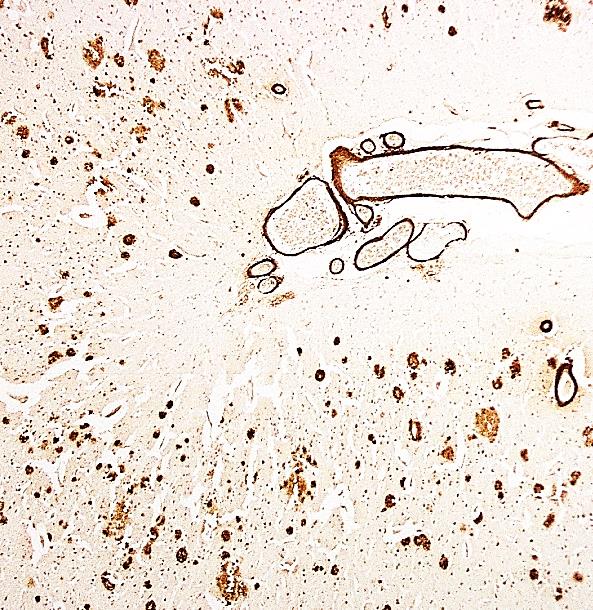

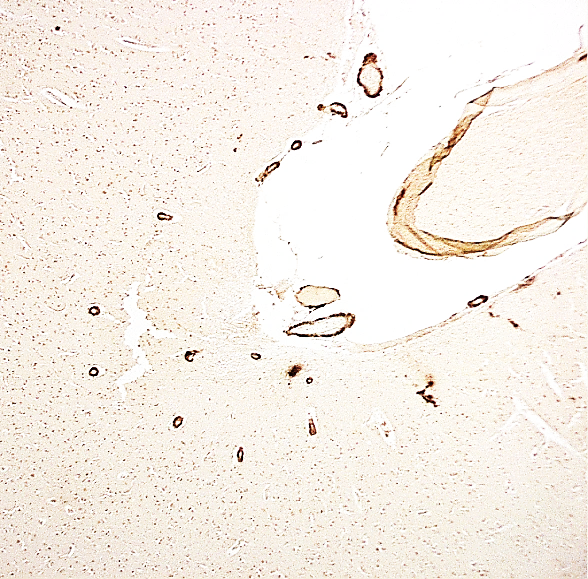


**250 µm**

**f**

**e**

**d**

**c**

**b**

**a**

Supplement: Supplementary file 1 — Supplementary material 1 (DOCX 4649 kb) Supplementary Fig. 1. Representative examples of the different plaque and CAA scores. Panel a, plaque score = 0, CAA score = 2. Panel b, plaque score = 1, CAA score = 0. Panel c, plaque score = 2, CAA score = 3. Panel d, plaque score = 3, CAA score = 4. Panel e, plaque score = 3, CAA score = 1. Panel f, plaque score = 4, CAA score = 2. Immunoperoxidase-haematoxylin [file 401_2018_1866_MOESM1_ESM.docx]
